# The Efficacy of Selected Synthetic Insecticides and Botanicals against Fall Armyworm, *Spodoptera frugiperda*, in Maize

**DOI:** 10.3390/insects10020045

**Published:** 2019-02-01

**Authors:** Birhanu Sisay, Tadele Tefera, Mulatu Wakgari, Gashawbeza Ayalew, Esayas Mendesil

**Affiliations:** 1 School of Pant Sciences, Haramaya University, P.O. Box 138, Dire Dawa, Ethiopia; birhanusisay291@gmail.com (B.S.), mulatuwakgari@yahoo.com (M.W.); 2 International Center of Insect Physiology & Ecology (icipe), P.O. Box 5689, Addis Ababa, Ethiopia; 3 Melkasa Agricultural Research Centre, P.O. Box 436, Adama, Ethiopia; gashawbeza@yahoo.com; 4 College of Agriculture & Veterinary Medicine, Jimma University, P.O. Box 307, Jimma, Ethiopia; emendesil@yahoo.com

**Keywords:** bioassay, cereal, fall armyworm, integrated pest management, invasive pest

## Abstract

Fall armyworm (FAW) was reported for the first time in Africa in 2016. FAW is widely distributed in Ethiopia, causing significant damage to maize. Nine synthetic insecticides belonging to different chemical groups and 11 pesticidal plants (botanicals) were tested for their efficacy against FAW under laboratory, greenhouse, and field conditions. In the laboratory, Radiant, Tracer, Karate, and Ampligo caused over 90% larval mortality 72 h after application. Malathion had moderate activity, causing 51.7% mortality 72 h after application, while Carbaryl was less effective, causing 28% mortality 72 h after application. In the greenhouse experiment, all synthetic insecticides reduced foliar damage to maize compared to the untreated control. Chemical sprays did not affect plant height, stem thickness, or leaf number. The highest fresh weight (471 g) was obtained from plants treated with Radiant. Among the botanicals tested, *Azadirachta indica*, *Schinnus molle*, and *Phytolacca dodecandra* resulted in the highest percentage larval mortality (>95%) 72 h after application. In the field, non-treated control plants showed extensive leaf injury compared to the synthetic insecticide- and botanical-treated plants. The synthetic insecticides and botanicals that showed high efficacy against FAW larvae can be used as components for integrated pest management (IPM) plans for FAW under smallholder farmer conditions in Ethiopia and elsewhere in Africa.

## 1. Introduction

Fall armyworm (FAW), *Spodoptera frugiperda* (JE Smith) (Lepidoptera: Noctuidae), is native to tropical and subtropical regions of the Americas and is the key insect pest of maize in tropical regions. The occurrence of FAW was reported in Africa for the first time in late 2016 in West Africa [1,2]. Subsequently, FAW has rapidly spread throughout Sub-Saharan Africa (SSA) and, currently, its occurrence has been confirmed in 44 African countries [3]. FAW is a highly polyphagous insect pest that attacks more than 80 plant species, including maize, sorghum, millet, sugarcane, and vegetable crops [4]; nevertheless, maize is the main crop affected by FAW in Africa. Given the importance of maize in Africa as a primary staple food crop, the recent invasion of FAW threatens the food security of millions of people in a region that will likely have an aggravated drought due to climate change/El Nino in SSA [3,4]. According to a recent estimate, in the absence of control methods, FAW has the potential to cause losses of an estimated 8.3 to 20.6 m tons of maize per annum (valued at US$2481–6187 m) in 12 maize-producing countries in SSA, which accounts for approximately 20% of the total production in the region [2].

FAW larvae cause damage to the plant by consuming foliage. Young larvae mainly feed on epidermal leaf tissue and also make holes in leaves, which is the typical damage symptom of FAW. Feeding on young plants through the whorl causes deadheart. In older plants, the larger larvae in the whorls can feed on maize cob or kernels, reducing yield and quality [2,5]. As is common with other major agricultural pests, the primary management strategy for FAW in the Americas is the use of synthetic insecticide sprays and genetically modified crops (Bt maize). Nevertheless, FAW has developed resistance to several synthetic insecticides [2,6], for example, according to Abrahams et al. [2], in the Americas FAW resistance has been reported to mode-of-action categories 1A (Carbamates) 1B (Organophosphates), and 3A (Pyrethroids-Pyrethrins). Furthermore, FAW resistance to Bt maize has been reported in different regions such as Puerto Rico, Brazil, Argentina, and the southeastern mainland USA [4]. This suggests the need for an integrated management strategy for the sustainable control of this invasive pest.

Since the occurrence of FAW in African countries, synthetic insecticides have been widely used as an emergency response to slow the spread of the pest and minimize damage to maize fields. Although synthetic insecticides play an important role in FAW management, given confirmed reports of the development of insecticide resistance in FAW populations [6] as well as other adverse effects due to the sole dependence on synthetic insecticide, it is imperative to use an integrated pest management strategy for FAW. Currently there are no registered synthetic insecticide for FAW control in African countries, except applications allowed through an emergency label, suggesting an urgent need for synthetic insecticide screening. Farmers have complained that the currently used synthetic insecticides are not effective against FAW; hence, they are forced to use high doses with frequent applications, which will lead to the accumulation of pesticides in the environment and speed up resistance development.

Botanical extracts have long been proposed as attractive alternatives to synthetic insecticides for pest management. Botanical extracts are eco-friendly, economical, usually target-specific, and biodegradable. The greatest strength of botanical extracts is their specificity, as most are essentially nontoxic and non-pathogenic to animals and humans [7,8]. Various plant species have shown insecticidal properties against FAW, for example extracts of neem, *Azadirachta indica* [9], *Argemone ochroleuca* Sweet (Papaveraceae) [10], Boldo, *Peumus boldus* Molina (Monimiaceae) [9], jabuticabeira, *Myrciaria cauliflora* [Mart.] O. Berg (Myrtaceae) [11]. Botanicals are cheap, readily available, and affordable, which are important qualities of pest control products for smallholder farmers in Africa [8]. The objectives of this study, therefore, are to evaluate selected synthetic insecticides against FAW under laboratory, greenhouse, and field conditions and test the efficacy of locally available insecticidal botanicals for the control of FAW larvae under laboratory and field conditions.

## 2. Materials and Methods

### 2.1. Description of the Study Area

All trials in this study were conducted at the Melkassa Agricultural Research Centre (MARC) in the entomology laboratory, greenhouse, and at MARC field sites. MARC is in the Oromia Regional State of Ethiopia, approximately 82 km east of the capital city, Addis Ababa. The center is located at 8°24′ N latitude, 39°21′ E longitude, and 1550 m above sea level.

### 2.2. Laboratory Bioassay of Synthetic Insecticides against FAW

Insect colony: A FAW starter colony was collected from an unsprayed maize farm at MARC. Approximately 100 fourth-instar larvae were collected; and the larvae were placed individually to avoid cannibalism into ventilated plastic jars (approximately 1 liter) in the lab and fed with maize leaves collected from 15–30-day-old maize plants, variety “Melkass 2”. The pre-pupal stage was transferred to a plastic jar one-third filled with soil for pupation. The pupae were collected and placed in a moistened Petri dish in an oviposition cage. Sterile cotton soaked in a sugar solution was placed in a Petri dish inside the oviposition cage as a food source for the emerging adults. The wall of the oviposition cage was lined with wax paper as an oviposition media. A photoperiod of 12 L:12D was maintained in the oviposition room. After approximately 2–3 days, old egg batches were collected from the oviposition cages and placed in sterile plastic jars. Eggs were monitored daily for hatching; as soon as the first instars emerged, they were provided with tender and fresh maize leaves. Rearing was performed at room temperature of 24–26 °C and 40–50% RH. Insects were reared as described above until a sufficient population was achieved to run the experiment. The second laboratory generation larvae were used for the present study.

Preparation of synthetic insecticides: Nine synthetic insecticides obtained from different sources were used (Table 1). These were: chlorantraniliprole (Coragen 200 SC), spinetoram (Radiant 120 SC), agro-Thoate 40% EC (Dimethoate 40%), spinosad (Tracer 480 SC), lambda-cyhalothrin (Karate 5 EC), Malathion 50% EC, chlorantrniliprole + lambda-cyhalothrin (Ampligo 150 SC), and Imidacloprid. Each synthetic insecticide was thoroughly mixed with water following the manufacturers’ recommendations for 5–10 min (Table 1).

Application of synthetic insecticides to maize: The maize variety “Melkass 2” was planted at the MARC field station on a plot size of 5 m × 5 m (25 m^2^), with a spacing of 75 cm between rows and 25 cm between plants within a row. Two seeds were planted per hill and were thinned to one seedling per hill two weeks after emergence. The maize plot was fertilized with diammonium phosphate (DAP) at planting at a rate of 100 kg/ha following standard agronomic practices for the area. Hand weeding was performed to control weeds. Four weeks after planting (at vegetative stage), each synthetic insecticide described above was applied to the maize plants using a backpack sprayer. For each synthetic insecticide, a separate plot of 5 m × 5 m maize was planted and sprayed.

Feeding larvae: The maize leaves were collected separately 1–2 h after being sprayed and were fed to the larvae. The collected leaves were cut and weighed to 60 g (from our FAW rearing experience, 45–60 g maize leaves can feed approximately 10 to 15 larvae for 2–3 days). Each 60 g contains 4–5 pieces of leaves of 5–6 cm in length. The leaves were placed in a plastic jar with a perforated lid. Ten third-instar larvae were released into the plastic jars containing the treated leaves. Leaves treated with sterile water were included as a control. The treatments were laid out in a completely randomized design (CRD) with nine replications. The insect diets (maize leaves) were changed every second day. The bioassay was repeated. Insect mortality was assessed 24, 48, and 72 h after treatment application. A larva was considered dead if it could not right itself after being placed on its dorsal surface.

### 2.3. Evaluation of Synthetic Insecticides against FAW in Greenhouse

Maize planting: Maize variety “Melkassa 2” was planted in plastic pots (30 L) in a greenhouse at MARC. The pots were filled with soil up to 15 cm from the top edge at a ratio of 2:1:1 top soil, compost, and sand soil, respectively. Five seeds were sown per pot. The pots were watered as required. Twenty days after seedling emergence, each plant was infested with five third-instar larvae. The larvae were obtained from a laboratory colony maintained at the MARC Entomology laboratory, as described above.

Insecticide application: The nine synthetic insecticides tested in the lab were used in the greenhouse trial. Each synthetic insecticide was thoroughly mixed with water following the manufacturers’ recommendations for 5–10 min. Pots with maize seedlings infested with larvae as described above were arranged in an area of 5 m × 5 m (25 m^2^) in the greenhouse. The amount of synthetic insecticides required to spray 25 m^2^ was calculated and calibrated. Applications were mixed with 500 mL of water and the solution was sprayed until the sprayer was empty. Each synthetic insecticide solution was added to a backpack sprayer and sprayed on the plants in pots. Plants treated with sterile water were included as a control. Application of insecticide spray was performed two times: seven days after infestation of plants with larvae and 14 days after infestation. The experiment was arranged in a completely randomized design (CRD) with four replications.

Data collected: Seven days after each of the synthetic insecticide applications, the numbers of live larvae, dead larvae, and pupae were counted in the treated plants and untreated control plants. It was not possible to recover all insects from the infested plants. Similarly, the FAW damage severity was recorded on an individual plant basis at seven-day intervals based on a rating scale described by Davis et al. [12] and Williams et al. [13]; 0 = no visible leaf damage, 1 = only pin-hole damage to the leaves, 2 = pin-hole and shot-hole damage to leaves, 3 = small elongated lesions (5–10 mm) on 1–3 leaves, 4 = midsized lesions (10–30 mm) on 4–7 leaves, 5 = large elongated lesions (>30 mm) or small portions eaten on 3–5 leaves, 6 = elongated lesions (>30 mm) and large portions eaten on 3–5 leaves, 7 = elongated lesions (>30 cm) and 50% of leaf eaten, 8 = elongated lesions (30 cm) and large portions eaten on 70% of leaves, 9 = most leaves have long lesions and complete defoliation is observed. Plant height, stem thickness, leaf number, fresh weight, and dry weight were recorded 70 days after planting. The dry weight was obtained after oven-drying the plant stems and leaves for 48 h at 70 °C.

### 2.4. Laboratory Bioassay of Botanicals against FAW

Plant extracts: 11 insecticidal plants/botanicals were collected from different parts of Ethiopia in July 2017. These were: *Azadirachta indica* (Neem), *Militia ferruginea* (Birbira), *Phytolacca dodecandra* (Endod), *Jatropha curcas* (Jatropha), *Schinnus molle*, *Croton macrostachyus* (Bisana), *Chenopodium ambrosoids* leaf extract, *Melia abyssinica* (Melia), *Eucalyptus globulus*, *Nicotina tabacum* (Tobacco), and *Lantana camara*. Leaf parts of *C. ambrosoids* and *N. tabacum*, and seeds of remaining plant species were dried separately under shade and then ground to a fine powder using a pestle and mortar. The powder of each botanical was soaked in distilled water at the effective rate previously reported by different authors for lepidopteran larvae. The rates were: 5 g of *A. indica* [14], 50 g of *M. ferruginea* [15], 11.5 g of *J. curcas* seed [16], 8 g of *M. abyssinica* [17], 35 g of *C. ambrosoids* leaf extract [18], 400 g *L. camara* seed extract [14,19], and 25 g of tobacco leaf, *E. globulus*, *P. dodecandra*, *C. macrostachyus*, and *S. molle* seed extracts [20]. The powder of each botanical plant was soaked in 100 mL of water for 24 h. Then, the powders of the different botanicals were filtered through a cheese cloth, and the solutions were left overnight.

Insects: FAW rearing was performed as described above, with newly molted third-instar larvae used for this bioassay.

Maize planting: The maize variety “Melkass 2” was planted at the MARC field station on a 5 m × 5 m plot with spacing of 75 cm between rows and 25 cm between plants within a row. Two seeds were planted per hill, thinned to one seedling per hill two weeks after emergence. The maize plot was fertilized with DAP at planting at the rate of 100 kg/ha application following standard agronomic practices for the area.

Application of botanical extracts: The 11 botanicals described above were screened against third-instar larvae. Approximately 60 g of maize leaf cuttings were prepared as described above and placed in a rectangular plastic box (4 cm height, 15 cm width, and 21 cm length) with a perforated lid using wire mesh to allow ventilation. Each box containing leaves was sprayed separately with 20 mL of each of the botanical extracts using a hand sprayer. Since there is no standard recommended volume of water for botanicals, we evaluated different volumes of water, and found that 20 mL provided uniform and adequate coverage. Ten third-instar larvae were released into each box containing the treated leaves. Leaves treated with sterile water were included as a control. The experiment was arranged in a complete randomized design (CRD) with four replications. The bioassay was repeated twice. Insect mortality was assessed 24 h, 48 h, and 72 h after treatment application. A larva was considered dead if it could not right itself after being placed on its dorsal surface. 

### 2.5. Field Evaluation of Selected Synthetic Insecticides and Botanicals Extracts against FAW

Maize variety “Melkass 2” was planted at the MARC field station on a plot size of 6 m x 4 m, with a spacing of 75 cm between rows and 25 cm between plants. The maize plot was fertilized with DAP at planting, at a rate of 100 kg/ha application. Manual weeding and irrigation was carried out when necessary. 

Preparation of synthetic insecticides: Three synthetic insecticides obtained from different sources were used. These were: lambda-cyhalothrin (Karate 5 EC) 320 mL/ha; spinetoram (Radiant 120 SC) 130 mL/ha and chlorantrniliprole + lambda-cyhalothrin (Ampligo 150 SC) 300 mL/ha. Each synthetic insecticide was thoroughly mixed with water following the manufacturers’ recommendations for 5–10 min.

Plant extracts: Three insecticidal plants/botanicals were collected from different parts of Ethiopia in May 2018. These were: *A. indica*, *P. dodecandra*, and *S. molle*. The seed part of the fruit of these plants were dried separately under shade and then ground to a fine powder using a pestle and mortar. The powder of each botanical was soaked in distilled water at a rate of 50 gm/L. Newly prepared powders were used in each application.

Treatment application: The treatments were applied using a knapsack sprayer at seven-day intervals starting at 25 days after planting and repeated at 32 and 39 days after planting. After spraying each treatment, the sprayer was rinsed with liquid soap once and then washed with water. The control plots were not sprayed. Randomized Complete Block Design (RCBD) with three replications was used for the experiment.

Before each spraying, both destructive (number of live larvae per plant) and non-destructive samples (leaf damage score) were taken. FAW leaf damage severity was recorded at seven-day intervals based on the rating scale described by Davis et al. [12] and Williams et al. [13] (see Section 2.3 for description).

### 2.6. Rotational Efficacy of Selected Synthetic Insecticide and Pesticidal Plants against FAW

These experiments were conducted at MARC (see Section 2.1 for description). Maize planting was done as indicated in Section 2.5. *Azadirachta indica* seed extract: The seed part of *A. indica* collected from Dire Dewa, Eastern Ethiopia was dried under shade and then ground to a fine powder using a pestle and mortar. The powder was soaked in distilled water at the rate of 50 gm/L.

Treatments: Treatments were applied either alone or in rotation for three applications at seven-day intervals. The experiment consisted of five treatments: (T1) *A. indica* alone sprayed for three applications; (T2) Karate 5 EC alone sprayed for three applications; (T3) *A. indica* sprayed in the first and second applications with Karate 5 EC sprayed in the third application; (T4) *A. indica* sprayed in the first application with Karate 5 EC sprayed in the second and third application; (T5) *A. indica* sprayed in the first application with Karate 5 EC sprayed in the second application; (T6) Unsprayed check (Table 2). The treatments were applied using a knapsack sprayer at seven-day intervals starting 25 days after planting. After spraying each treatment, the sprayer was rinsed with liquid soap once and then washed with water. The control plots were not sprayed. Randomized Complete Block Design (RCBD) with three replications was used for the experiment.

Before each spray application, destructive sample was performed to evaluate the number of live larvae per plant and non-destructive leaf scores were taken every seven days using the Davis et al. [12] and Williams et al. [13] scale as previously described (see Section 2.3).

### 2.7. Statistical Analysis

Mean percentage larval mortality, plant height, stem thickness, leaf number, fresh weight, dry weight, and mean numbers of FAW larvae obtained from laboratory, greenhouse, and field trials were subjected to one-way analysis of variance (ANOVA) using a generalized linear model. Field trials were arranged in a randomized block design. The percent larval mortality from laboratory bioassays of synthetic insecticides and botanicals was transformed using an arcsine transformation to normalize the variances [21]. The significance level was set at 0.05, and the means were separated by Tukey’s honest significant difference test. Leaf damage score data were categorical variables and were analyzed using the Kruskal–Wallis test. All statistical analyses were performed using the MINITAB 16 statistical software.

## 3. Results

### 3.1. Laboratory Bioassay of Synthetic Insecticides against FAW

There were significant differences among the synthetic insecticides in causing mortality to larvae at 24 h (F = 41.69; df = 9; *p* < 0.001), 48 h (F = 52.6; df = 9; *p* < 0.001), and 72 h (F = 74.7; df = 9; *p* < 0.001) after treatment application (Table 3). Karate 5 EC caused 77.8% mortality, followed by Ampligo 150 SC (62.2% mortality), Radiant 120 SC (61.1% mortality), and Coragen 200 SC (60% mortality). Radiant 120 SC caused the highest mortality of 96.7% 48 h after treatment application and 100% mortality 72 h after treatment application, while Karate 5 EC caused 96.7% mortality 48 h and 72 h after treatment application. Malathion was moderate in causing 51.7% mortality 72 h after treatment application, while Carbaryl was less effective, causing 28% mortality.

### 3.2. Evaluation of Synthetic Insecticides against FAW in Greenhouse

Mean mortality of FAW larvae was significantly different among treatments during the first- (F = 4.28; df = 9; *p* = 0.003) and second-round spraying (F = 4.85; df = 9; *p* = 0.002) (Table 4). Dimethoate 40% caused the highest larval mortality (40%), followed by Coragen 200 SC (mortality 33.3%), Radiant 120 SC (mortality 33.3%), Karate 5 EC (mortality 33.3%), Tracer 480 SC (mortality 20%), and Carbaryl (mortality 6.7%). Malathion caused no mortality during the first spray. During the second round of synthetic insecticide spraying, Karate 5 EC caused 60% mortality, followed by Dimethoate 40%, causing 53.3% larval mortality. Radiant 120 SC, Ampligo 150 SC, and Imidacloprid caused 40% mortality. Carbaryl and Malathion were the least effective, causing 6.7% larval mortality (Table 4). It was not possible, however, to recover all the insects from the infested plants; hence, mortality did not add up to 100%.

**Figure 1:**
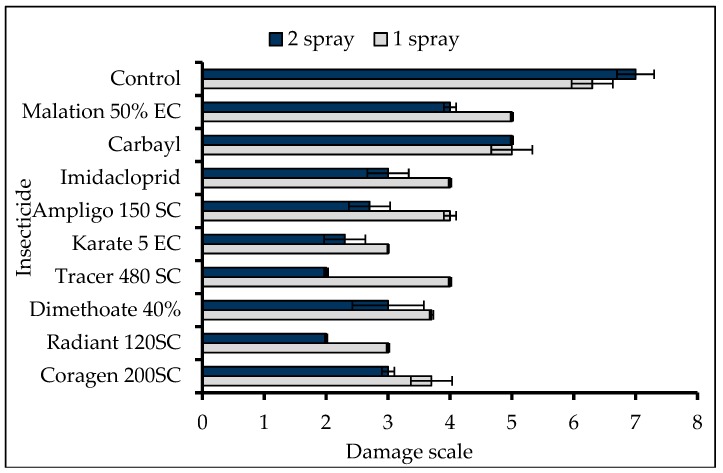
Mean (± SEM) leaf damage of maize by FAW under different treatments in the greenhouse.

Leaf damage inflicted by FAW larvae was significantly different among treatments in both first (H = 23.96; df = 9; *p* = 0.004) and second-round spraying (H = 24.31; df = 9; *p* = 0.004). The non-treated control plants had extensive leaf injury by FAW larvae compared to the plants treated with synthetic insecticides (Figure 1). In the first-round spraying, the lowest leaf damage [3] was recorded in plants treated with Radiant 120 SC and Karate 5 EC, whereas in the second-round spraying, Radiant 120 SC and Tracer 480 SC showed the lowest leaf damage (Figure 1). Plant height, stem thickness, leaf number, and dry matter of maize plants showed no significant differences among treatments. On the other hand, there were significant differences in the fresh weight (F = 3.16; df = 9; *p* = 0.015) among treatments. The highest fresh weight (471 g) was obtained from plants treated with Radiant 120 SC (Table 5).

### 3.3. Laboratory Bioassay of Botanicals against FAW

There were significant differences between botanicals in causing mortality to larvae (Table 6). Extracts of *P. dodecandra*, *S. molle*, *A. indica*, and *J. curcas* caused the highest percentage mortality, 56.7 to 63.3%, to the larvae (F = 55.94; df = 11; *p* < 0.001) 24 h after treatment application and 80 to 90% mortality (F = 54.04; df = 11; *p* < 0.001) 48 h after treatment application. *Phytolacca dodecandra*, *S. molle*, and *A. indica*, however, caused over 96% larval mortality 72 h after treatment application. *Melia abyssinica* and *J. curcas* resulted in a higher percentage larval mortality (>90%) 72 h after treatment application; however, *E. globulus* and *C. ambrosoids* caused the lowest mortality, 8–20%.

### 3.4. Field Evaluation of Selected Synthetic Insecticide and Botanical Extracts against FAW

**Figure 2:**
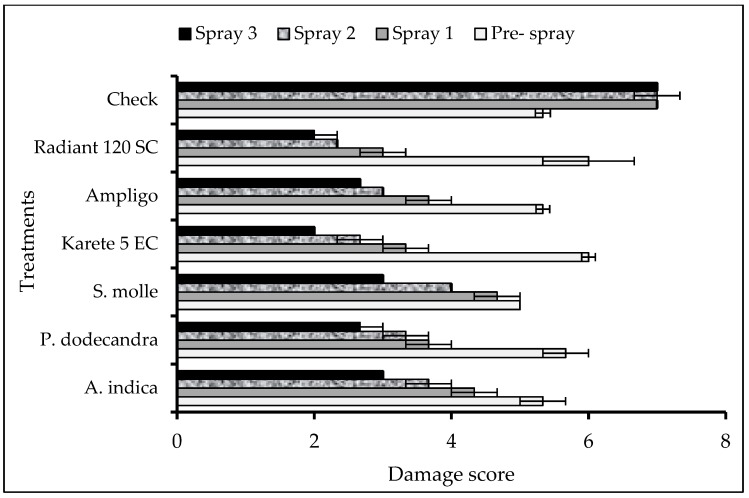
Mean (± SEM) of leaf damage of maize by FAW under different treatments in the field.

Leaf damage inflicted by FAW larvae was significantly different among treatments in both first (H = 14.68; df = 5; *p* = 0.012) and second-round spraying (H = 11.39; df = 5; *p* = 0.044). The non-treated control plants had extensive leaf injury by FAW larvae compared to the synthetic insecticide- and botanical-treated plants. In the first-round spraying, the lowest leaf damage was recorded in plants treated with Karate 5 EC and Radiant 120 SC, and similar results were obtained in the second- and third-round sprayings (Figure 2). The number of live larvae in treated plants was lower compared to non-treated plants. In the first-round spraying, the lowest number of live FAW larva was recorded in the Radiant-treated plants (F = 33.73; df = 6; *p* < 0.001). The number of live larvae was significantly reduced in treated plants in the second (F = 28.7; df = 6; *p* < 0.001) and third (F = 99.43; df = 6; *p* < 0.001) sprayings. In the second- and third-round sprayings Karate 5 EC, Radiant 120 SC and *A. indica* showed the lowest number of live larvae, while no live larvae were recorded from plants sprayed with Radiant 120 SC, Karate 5 EC, and *A. indica* in the second- and third-round sprayings (Table 7).

### 3.5. Application of Selected Insecticides and Botanical Extracts in Rotation against FAW

**Figure 3:**
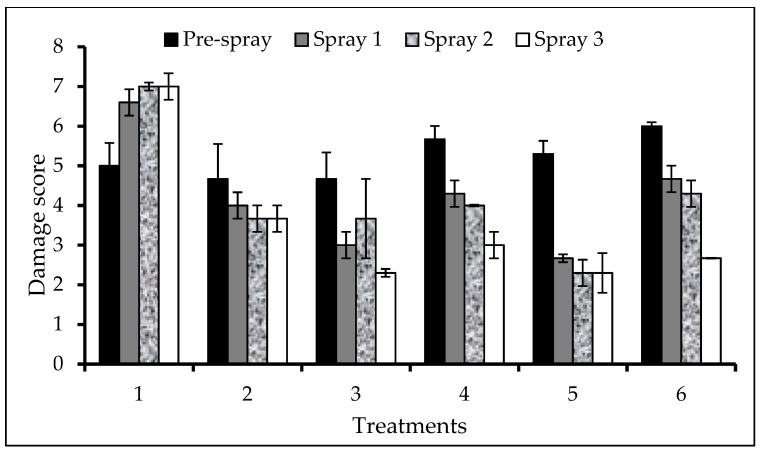
Mean (± SEM) of leaf damage of maize by FAW under different treatments in the field. Note: (T1) *A. indica* alone sprayed for three applications; (T2) Karate 5 EC alone sprayed for three applications; (T3) *A. indica* sprayed in the first and second applications with Karate 5 EC sprayed in the third application; (T4) *A. indica* sprayed in the first application with Karate 5 EC sprayed in the second and third application; (T5) *A. indica* sprayed in the first application with Karate 5 EC sprayed in the second round; (T6) Unsprayed Control.

Leaf damage caused by FAW larvae was not significantly different among treatments in all the spray rounds (Figure 3).

The numbers of live larvae were reduced after sprayed with different treatments compared to untreated check (unsprayed plants). In the first round of spraying, except for plants treated with *A. indica* + Karate 5 EC (T3), all the treatments showed significantly (F = 23.88; df = 5; *p* < 0.001) lower numbers of live larvae. The number of live larvae were significantly lower in all treated plants in the second (F = 7.35; df = 5; *p* = 0.003) and third (F = 7.5; df = 5; *p* = 0.002) round sprays as compared with the check (unsprayed plants) (Table 8). Furthermore, no live larvae were recorded from plants sprayed with Karate 5 EC (T2) in the second-round spraying, while about one larva was recorded in all treated plants in the third-round spraying, with the exception of plants treated with *A. indica* + Karate 5 EC (T3).

## 4. Discussion

In this study, all of the synthetic insecticides tested were toxic to FAW larvae, and some synthetic insecticides demonstrated high larval mortality in both laboratory and greenhouse trials. In laboratory bioassays, moderate to high larval mortality was achieved with Karate 5 EC, Tracer 480 SC, Coragen 200 SC, Ampligo 150 SC, and Radiant 120 SC. It was noted that in both the laboratory and greenhouse trials, the percent larval mortality increased over time after synthetic insecticides application, which may indicate residual toxicity of the synthetic insecticides to FAW. The results obtained in the greenhouse study demonstrated a significant reduction in leaf damage to maize compared to the control, which is attributed to the reduced number of larvae in treated plants. Consequently, the highest fresh and dry weights were obtained from plants treated with synthetic insecticides compared to unsprayed control plants. 

As is common with other insect pest species, synthetic insecticides are important management options in FAW control in the Americas [22]. In Mexico, chemical control of FAW in maize is achieved by the application of methyl parathion, chlorpyrifos, methamidophos, and phoxim, among other synthetic insecticides [23]. In the southern United States, synthetic insecticides are applied on sweetcorn against FAW, often 3–4 times weekly in much of the southeast. In Florida, FAW is one of the most important sweetcorn pests, and synthetic insecticides are applied against FAW to protect both the vegetative stages and reproductive stage of corn [5]. Several insecticide applications are required to kill larvae feeding deep in the whorl of plants. In situations in which overhead sprinklers are used for irrigation, synthetic insecticides can also be applied in the irrigation water. Keeping plants free of larvae during the vegetative period can help to reduce the number of sprayings needed at the silking stage [24]. Some of the synthetic insecticides reported by those authors corroborate the findings of the present study. For example, Belay et al. [25] reported >60% FAW mortality 16 h after application of Radiant, Orthene, and Larvin. In another study, Intrepid 2F, Lannate 2.4LV, Sevin XLR Plus 4F, and Tracer 4SC effectively reduced FAW larvae under field conditions [26]. Hence, sprayings should be spaced evenly during the growing period instead of concentrated at the silking period. 

Although synthetic insecticides are effective to control FAW, in Africa the increased risk to human health due to a lack of appropriate safety precautions is a major concern about synthetic insecticide use [2]. Furthermore, the development of resistance to major classes of synthetic insecticides in native regions of this pest [6] is another problem. This suggests the need for resistance management as a vital component of IPM. Resistance management is likely to be successful when combined with routine monitoring of pests, use of reasonable treatment thresholds, and the full use of non-pesticidal methods, such as biological and cultural control, field sanitation, and host plant resistance. Judicious and appropriate use of synthetic insecticides is essential for the successful management of FAW and to sustain the increased productivity of maize in Africa.

The recent invasion of FAW has alarmed governments of numerous African countries and caused them to deploy a massive pesticide spraying program as an emergency response in FAW-affected areas, mainly to maize fields to protect against crop damage and prevent the expansion of the pest. In recent surveys conducted in Kenya and Ethiopia, it has been noted that farmers are applying different types of unregistered synthetic insecticides [27], possibly because of the invasive nature of the pest, which requires a rapid response and a lengthy pesticide registration process.

In the present study, locally available insecticidal plants showed different levels of efficacy against FAW larvae. Extracts of *A. indica*, *P. dodecandra*, and *S*. *molle* consistently resulted in high larval mortality. In line with the present study, Silva et al. [9] reported high larval mortality of FAW using a seed cake extract of *A. indica*. In recent studies, ethanolic extracts of *A. ochroleuca* Sweet (Papaveraceae) showed FAW larval mortality due to a reduction in feeding and slow larval growth [10]. In other studies, Boldo and *P. boldus* Molina caused toxicity by acting as a feeding inhibitor and showed repellent properties at high concentration [9]. Extracts of *Cedrela salvadorensis* and *C. dugesii* caused larval mortality [28]. Alves et al. [11] also observed that extracts of jabuticabeira and *M. cauliflora* [Mart.] O. Berg caused larval mortality and an increase in the duration of larval and pupal stages, respectively. These studies demonstrated the potential of using insecticidal plants as a component of an integrated pest management program, mainly for smallholder farmers. These plants are locally grown in many parts of Africa and can be used by small-scale farmers wherever available as an alternative approach to FAW management. Furthermore, the use of botanicals by small-scale farmers is not a regulatory issue in many developing countries. A recent review by Stevenson et al. [8] reported a huge diversity of pesticidal plants in Africa and prospects for developing plant-based pesticides.

FAW is likely to directly affect capital costs through increased labor requirement and the increased level of IPM knowledge required to address the pest; through yield losses and the ability of agricultural lands to respond to shocks; and financially, through the increased cost of production due to cost of control and its effect on income. The arrival of FAW in Africa creates a new risk for countries that import crops from affected African countries if FAW is absent from the importing country, including countries in Asia and Europe [2]. The occurrence of multiple generations, ability to migrate, and ability to feed on a wide range of host plants makes FAW one of the most difficult pests to control in Africa. FAW poses a new threat to food security on the continent. Quick and coordinated action, enormous awareness creation, technological innovation, and national, regional, and international collaboration are required to tackle the menace of the FAW pest to avoid economic adversity for smallholder farmers in Africa [29]. Development and deployment of an effective integrated pest management strategy, which can provide sustainable solutions to effectively tackle the adverse effects of FAW, is required. The current study, therefore, contributes to the management of the FAW in screening effective pesticides and botanical plants.

## 5. Conclusions

From the present study, it was observed that application of the synthetic insecticides Karate 5 EC, Coragen 200 SC, Radiant 120 SC, Dimethoate 40%, Tracer 480 SC, and Ampligo 150 SC was effective and significantly increased FAW larval mortality, reduced leaf damage, and increased biomass in maize compared to the untreated control. Among the botanicals, *A. indica*, *P. dodecandra*, and *S. molle* had the highest efficacy in terms of causing the highest mortality to FAW larvae. In field experiments, no live larvae were recorded from plants sprayed with Radiant, Karate 5 EC, or *A. indica*. In addition, in the combination of treatments, no live larvae were recorded from plants sprayed with Karate 5 EC in the second-round spraying, while less than one larva was recorded in all treated plants after the third round of spraying, with the exception of plants treated with *A. indica* + Karate 5 EC. The most effective pesticides and botanicals are therefore recommended for the management of FAW in maize. However, an IPM approach is needed to control FAW. Reliance on chemical control alone may, in the long run, increase the likelihood of FAW resistance to insecticides.

## Figures and Tables

**Table 1:** List of synthetic insecticides, their active ingredients (a.i.), and suggested label rate used in the experiment against FAW.

Trade Name	Active Ingredient	Manufacturer	Insecticide/ha
Coragen^®^ 200 SC	chlorantraniliprole	DuPont	250 mL
Radiant^®^ 120 SC	Spinetoram	Dow Agro Sciences	130 mL
Dimethoate 40%	agro-Thoate 40% EC	Adami Tuluu	1.5 L
Tracer 480 SC	Spinosad	Dow Agro Sciences	150 mL
Karate 5 EC	lambda-cyhalothrin	Syngenta	320 mL
Ampligo 150 SC	chlorantrniliprole + lambda-cyhalothrin	Syngenta	300 mL
Imidacloprid SL		Tagror	112.5 mL
Carbaryl WP		Honobor Weilike	2 kg
Malathion 50% EC		Honobor Weilike	2 L

**Table 2:** Details of different treatments used in the experiment.

Treatment	Plant Extracts/Insecticides	Spray Rotation		
		1st	2nd	3rd
1	*A. indica*	*	*	*
2	Karate 5 EC	*	*	*
3	*A. indica*	*	*	-
	Karate 5 EC	-	-	*
4	*A. indica*	*		
	Karate 5 EC	-	*	*
5	*A. indica*	*	-	-
	Karate 5 EC		*	-
6	Unsprayed Control			

* Sprayed; - not sprayed.

**Table 3:** Mean percentage (± SEM) of cumulative mortality of FAW larvae at 24, 48, and 72 h after application of synthetic insecticides in a laboratory test.

Treatments	Percent Larval Mortality (± SEM)		
	24 h	48 h	72 h
Coragen 200 SC	60 ± 5.59 ab	85 ± 5.46 abc	87.5 ± 5.20 abc
Radiant 120 SC	61.1 ± 4.77 ab	96.7 ± 1.18 a	100 ± 0 a
Dimethoate 40%	35 ± 5.59 c	73.3 ± 6.35 bc	77.5 ± 3.82 bc
Tracer 480 SC	55 ± 8.29 bc	84.4 ± 7.52 ab	95 ± 2.24 a
Karate 5 EC	77.8 ± 2.90 a	96.7 ± 1.67 a	96.7 ± 1.67 a
Ampligo 150 SC	62.2 ± 3.83 ab	87.8 ± 3.64 ab	92.5 ± 3.23 ab
Imidacloprid	40 ± 5.71 bc	63.3 ± 4.64 c	70.83 ± 5.07 cd
Carbaryl	3.3 ± 1.18 d	13.9 ± 2.47d	28.3 ± 1.86 e
Malathion 50% EC	8.3 ± 2.2 d	32.8 ± 5.34 d	51.7 ± 7.71 d
Control	0.0b ± 0 d	0.0b ± 0 d	1.1 ± 0.735 f
ANOVA			
*F* =	41.69	52.82	74.72
*DF* =	9	9	9
*p* <	0.001	0.001	0.001

Means within a column not sharing a common letter are significantly different at *p* < 0.05 using Tukey’s test.

**Table 4:** Mean percentage (± SEM) FAW larval mortality at two different spray times in the greenhouse.

Synthetic Insecticides	1st Spray	2nd Spray
	Larval Mortality	Larval Mortality
Coragen 200 SC	33.3 ± 4.23 ab	46.7 ± 6.67 abc
Radiant 120 SC	33.3 ± 4.23 ab	40.0 ± 0 abc
Dimethoate 40%	40.0 ± 0 a	53.3 ± 6.67 ab
Tracer 480 SC	20.0 ± 11.5 ab	26.7 ± 6.67 abc
Karate 5 EC	33.3 ± 4.23 ab	60.0 ± 11.5 a
Ampligo 150 SC	13.3 ± 8.74 ab	40.0 ± 11.5 abc
Imidacloprid	26.7 ± 13 ab	40.0 ± 23.1 abc
Carbayl	6.7 ± 8.74 ab	6.7 ± 6.67 bc
Malathion 50% EC	0.0 ± 0 b	6.7 ± 6.67 bc
Control	0.0b ± 0 b	0.0 ± 0b c
ANOVA		
*F* =	4.28	4.85
*DF* =	9	9
*p* =	0.003	0.002

Means within a column not sharing a common letter are significantly different at *p* < 0.05 using Tukey’s test.

**Table 5:** Mean percentage (± SEM) of plant height, stem thickness, leaf number, fresh and dry weight of maize under different synthetic insecticide treatments in the greenhouse.

Treatments	PH	ST	LN	FW	DW
Coragen 200 SC	161.7 ± 3.33	19.0 ± 1.25	13.7 ± 0.67	334 ± 25.8 ab	65 ± 10.1
Radiant 120SC	172.3 ± 3.33	22.0 ± 2.13	15.0 ± 1.00	471 ± 43.9 a	46 ± 14.1
Dimethoate 40%	161.7 ± 3.33	18.5 ± 1.45	13.3 ± 0.88	326 ± 22.4 ab	77 ± 16.5
Tracer 480 SC	170.0 ± 17.6	19.5 ± 2.13	14.3 ± 1.20	396 ± 49.0 ab	95 ± 14.6
Karate 5 EC	170.7 ± 13.7	20.4 ± 0.20	14.7 ± 0.88	399 ± 28.2 ab	74 ± 6.06
Ampligo 150 SC	165.7 ± 8.33	19.5 ± 1.10	13.7 ± 0.33	373 ± 37.6 ab	54 ± 7.91
Imidacloprid	148.3 ± 10.1	18.1 ± 1.64	13.0 ± 0.57	301 ± 13.6 ab	72 ± 7.44
Carbaryl	121.7 ± 3.33	17.2 ± 2.96	13.0 ± 0.00	270 ± 90.3 ab	59 ± 25.0
Malathion 50% EC	141.3 ± 28.8	17.5 ± 1.82	13.0 ± 0.57	249 ± 70.0 ab	46 ± 14.0
Control	112.3 ± 25.2	13.8 ± 1.92	12.3 ± 1.67	166 ± 20.3 b	22 ± 1.99
ANOVA					
*F* =	2.08	1.48	0.89	3.16	2.37
*DF* =	9	9	9	9	9
*p* =	0.083	0.220	0.552	0.015	0.052

Means within a column not sharing a common letter are significantly different at *p* < 0.05 using Tukey’s test. PH = plant height; ST = stem thickness; LN = leaf number; FW = fresh weight; DW = dry weight.

**Table 6:** Mean percentage (± SEM) of cumulative mortality of FAW larvae 24, 48, and 72 h after feeding on maize leaves treated with botanical extracts in the laboratory test.

Treatments	Percent Larval Mortality		
	24 h	48 h	72 h
*A. indica*	60.0 ± 0 a	90.0 ± 5 a	98.3 ± 1.67 a
*S. molle*	58.3 ± 6.67 a	80.0 ± 5 ab	96.7 ± 1.67 a
*M. abyssinica*	31.7 ± 8.82 b	63.3 ± 8.33 bc	90.0 ± 2.89 ab
*M. ferruginea*	20.0 ± 0b c	41.7 ± 1.67cd	78.3 ± 4.41 bc
*P. dodecandra*	63.3 ± 1.67 a	83.3 ± 6.01 ab	96.7 ± 3.33 a
*J. curcas*	56.7 ± 4.41 a	85.0 ± 2.89 ab	91.7 ± 1.67 ab
*C. macrostachyus*	8.3 ± 1.67 cd	36.7 ± 1.67 cd	75.0 ± 5.77 bc
*N. tabacum*	3.3 ± 1.67 de	21.7 ± 3.33 de	50.0 ± 2.89 cd
*L. camara*	5.0 ± 0 cde	18.3 ± 1.67de	40.0 ± 0 d
*E. globulus*	0.0 ± 0 e	1.7 ± 1.67 f	8.3 ± 3.33 ef
*C. ambrosoids*	1.7 ± 1.67de	5.0 ± 2.89 ef	21.7 ± 6.01 de
Untreated	0.0 ± 0 e	0.0 ± 0 f	1.67 ± 1.67 f
ANOVA			
*F* =	55.94	54.04	57.07
*DF* =	11	11	11
*p* <	0.001	0.001	0.001

Means within a column not sharing a common letter are significantly different at *p* < 0.05 using Tukey’s test.

**Table 7:** Mean number (± SEM) of FAW larvae after treatment application.

Treatments	Mean Numbers of Live Larvae			
	Pre-Spray	1st Spray	2nd Spray	3rd Spray
*A. indica*	3.0 ± 0.32 a	1.0 ± 0.0 ab	0.7 ± 0.0 a	0.0 ± 0.0 b
*P. dodecandra*	3.7 ± 0.26 a	1.7 ± 0.13 ab	1.3 ± 0.0 b	1.3 ± 0.0 c
*S. molle*	3.7 ± 0.26 a	3.0 ± 0.23 ab	2.0 ± 0.12 bc	1.7 ± 0.1 c
Karate 5 EC	5.7 ± 0.17 a	1.3 ± 0.1 ab	0.0 ± 0.0 a	0.0 ± 0.0 b
Ampligo 150 SC	4.3 ± 0.13 a	1.3 ± 0.1 ab	2.0 ± 0.0 bc	0.7 ± 0.0 b
Radiant 120SC	5.3 ± 0.24 a	0.3 ± 0.0b	0.0 ± 0.0 a	0.0 ± 0.0 b
Check	3.6 ± 0.37 a	3.7 ± 0.3a	2.7 ± 0.15 c	3.3 ± 0.15 a
ANOVA				
*F* =	0.87	33.73	28.7	99.43
*DF* =	6	6	6	6
*p* =	0.6	<0.001	<0.001	<0.001

Means within a column not sharing a common letter are significantly different at *p* < 0.05 using Tukey’s test.

**Table 8:** Mean number (± SEM) of FAW larvae after treatment application.

Treatments	Mean Numbers of Live Larvae (±SEM)			
	Pre-Spray	1st Spray	2nd Spray	3rd Spray
T1	3.0 ± 0.15 a	2.0 ± 0.1 bc	2.7 ± 0.3 b	0.3 ± 0.0 b
T2	3.7 ± 0.18 a	1.3 ± 0.1 c	0.0 ± 0.0 c	0.0 ± 0.0 b
T3	3.3 ± 0.27a	2.7 ± 0.15 ab	2.7 ± 0.1 b	1.7 ± 0.3 b
T4	4.0 ± 1.5 a	1.0 ± 0.0 c	0.7 ± 0.0 bc	0.0 ± 0.0 b
T5	4.0 ± 1.08 a	2.0 ± 0.0 b	1.0 ± 0.0 bc	0.3 ± 0.0 b
T6	5.0 ± 1.2 a	3.0 ± 0.15a	5.3 ± 0.6 a	5.3 ± 1.05 a
ANOVA				
*F* =	1.0	23.88	7.35	7.5
*DF* =	5	5	5	5
*p* =	0.57	0.00	0.003	0.002

Means within a column not sharing a common letter are significantly different at *p* < 0.05 using Tukey’s test. Note: (T1) *A. indica* alone sprayed for three applications; (T2) Karate 5 EC alone sprayed for three applications; (T3) *A. indica* sprayed in the first and second applications with Karate 5 EC sprayed in the third application; (T4) *A. indica* sprayed in the first application with Karate 5 EC sprayed in the second and third application; (T5) *A. indica* sprayed in the first application with Karate 5 EC sprayed in the second round; (T6) Unsprayed Control.
